# Crystal structure of an L chain optimised 14F7 anti-ganglioside Fv suggests a unique tumour-specificity through an unusual H-chain CDR3 architecture

**DOI:** 10.1038/s41598-018-28918-5

**Published:** 2018-07-18

**Authors:** Kaare Bjerregaard-Andersen, Hedda Johannesen, Noha Abdel-Rahman, Julie Elisabeth Heggelund, Helene Mykland Hoås, Fana Abraha, Paula A. Bousquet, Lene Støkken Høydahl, Daniel Burschowsky, Gertrudis Rojas, Stefan Oscarson, Geir Åge Løset, Ute Krengel

**Affiliations:** 1 Department of Chemistry, University of Oslo, NO-0315 Oslo, Norway; 2 Department of Biochemistry, Faculty of Pharmacy, Mansoura University, Mansoura, 35516 Egypt; 3 School of Chemistry, University College Dublin, Belfield, Dublin, 4 Ireland; 4 Centre for Immune Regulation and Department of Immunology, University of Oslo and Oslo University Hospital, NO-0372 Oslo, Norway; 5 Center of Molecular Immunology, Calle 216 esq 15, Atabey, Playa, La Habana, CP 11300 Cuba; 6 Department of Biosciences, University of Oslo, NO-0316 Oslo, Norway; 7 Nextera AS, NO-0349 Oslo, Norway; 8 Present Address: School of Biomedical Sciences, University of Leeds, Leeds, LS2 9JT UK; 9 Present Address: Leicester Institute of Structural and Chemical Biology, University of Leicester, Leicester, LE1 7HB UK

## Abstract

Targeted cancer immunotherapy offers increased efficacy concomitantly with reduced side effects. One antibody with promising clinical potential is 14F7, which specifically recognises the NeuGc GM3 ganglioside. This antigen is found in the plasma membrane of a range of tumours, but is essentially absent from healthy human cells. 14F7 can discriminate NeuGc GM3 from the very similar NeuAc GM3, a common component of cell membranes. The molecular basis for this unique specificity is poorly understood. Here we designed and expressed 14F7-derived single-chain Fvs (scFvs), which retained the specificity of the parent antibody. Detailed expression and purification protocols are described as well as the synthesis of the NeuGc GM3 trisaccharide. The most successful scFv construct, which comprises an alternative variable light chain (V_LA_), allowed structure determination to 2.2 Å resolution. The structure gives insights into the conformation of the important CDR H3 loop and the suspected antigen binding site. Furthermore, the presence of V_LA_ instead of the original V_L_ elucidates how this subdomain indirectly stabilises the CDR H3 loop. The current work may serve as a guideline for the efficient production of scFvs for structure determination.

## Introduction

Immunotherapy has emerged as a highly successful strategy in cancer treatment^1,2^. An array of different approaches ranging from classical intradermal vaccination, antibody-based immune checkpoint blockade to redirection of the patients’ own immune system are utilised^3^. However, a central and shared component of all these therapeutic avenues is the high degree of disease specificity conferred by focusing on targets over-expressed by or exclusive to the tumour tissue^4–6^. This selective targeting is most frequently offered by exploiting the inherent binding properties of antibodies (Abs) and T cell receptors as targeting units^7,8^. Despite showing very encouraging results in some cancers, such as leukemia, these immunotherapeutic approaches still lack efficacy in most solid tumours due to the challenging immunomodulatory mechanisms and scarcity of potent and safe targets^9–11^. Gangliosides are extracellularly exposed, sialic acid-containing glycosphingolipids located in the plasma membrane and potential target antigens for cancer immunotherapy^12,13^. One such antigen is the ganglioside NeuGc GM3, which contains the sialic acid *N*-glycolyl neuraminic acid. NeuGc GM3 is displayed on the cell surface of a range of cancer cells, *e.g*. breast carcinoma, melanoma, retinoblastoma and lymphoid tumors^14–17^. The ceramide tail is anchored in the membrane, whereas the hydrophilic glycan head is exposed to the extracellular environment. NeuGc GM3 is structurally highly similar to the ganglioside *N*-acetyl GM3 (NeuAc GM3), with the only difference being an additional oxygen atom found in NeuGc GM3. The presence of NeuGc GM3 in certain human cancer types remains enigmatic^18–20^. NeuAc GM3 can be converted to NeuGc GM3 by cytidine monophosphate-*N*-acetylneuraminic acid hydroxylase (cmah)^21^. However, in contrast to other mammals, humans carry a deletion in the *CMAH* gene rendering normal human cells unable to produce NeuGc GM3^22,23^. Nevertheless, there are minute amounts of NeuGc GM3 present even in human healthy cells, probably due to incorporation through the diet^24,25^. Still, NeuGc GM3 fulfils the criteria of being a tumour-specific extracellular marker, which makes it a very potent antigen for targeted cancer immunotherapy.

The monoclonal antibody (mAb) 14F7 is a murine IgG that binds selectively to NeuGc GM3^14^. It has been reported to kill cells by disrupting the integrity of tumour cells through an oncosis-like mechanism^26^ and in addition serves as a powerful prognostic tool^15,27^. The affinity for NeuGc GM3 has earlier been reported to be in the low nanomolar range (*K*_D_ = 25 nM), whereas 14F7 exhibits poor affinity for NeuAc GM3^28^. However, the structural basis for 14F7′s discriminatory power is currently poorly understood. The crystal structure of the 14F7 Fab domain without its ganglioside ligand was determined more than a decade ago^29^, but the ganglioside complex has remained recalcitrant to crystallisation and efforts to reliably model the binding mode have still left many open questions^30^. What is known from light chain shuffling studies^28^ is that the interaction is mainly with the 14F7 heavy chain Fv. Moreover, *in vitro* directed evolution through combinatorial phage display and subsequent mutagenesis have identified crucial residues for the recognition of the ganglioside^30^.

One of our main experimental challenges has been to reproducibly crystallise the 14F7 Fab. Therefore we wished to explore an alternative approach, based on a single-chain Fv (scFv). A scFv is an antibody-derived fragment that contains the heavy chain variable region (V_H_) and the light chain variable region (V_L_) tethered covalently by a linker. The construction and expression of a panel of 14F7-derived scFv antibody fragments that maintain recognition of NeuGc GM3 using a phagemid vector have been reported previously^28^, but only allowed the production of small amounts of soluble scFvs. For structural characterisation, we need larger quantities of stable protein. With the aim of obtaining the crystal structure of the NeuGc binding paratope, we set out to explore alternative scFv design strategies, and develop expression and purification protocols that may also be applicable to other systems. We describe the construct design and validated production protocols for four 14F7-derived scFvs and report the successful crystallisation and structure determination of one of these to 2.2 Å resolution. In addition we describe a synthesis protocol for the NeuGc GM3 trisaccharide. Together, this information provides a valuable platform for future structural investigation and engineering of the unique specificity of 14F7 for its tumour specific antigen.

## Results

### Synthesis of the NeuGc GM3 trisaccharide

**Figure 1:**
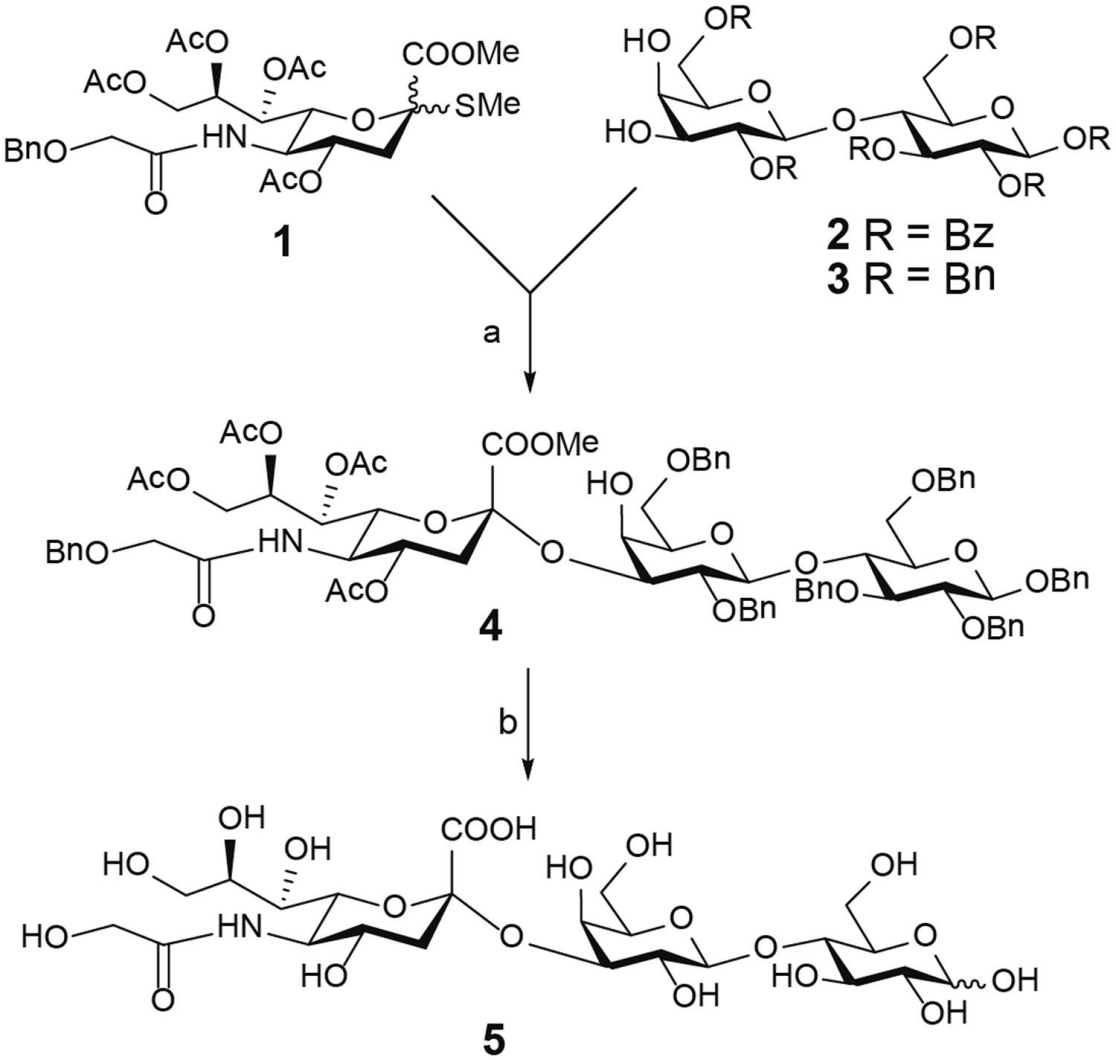
Reaction scheme for NeuGc GM3 trisaccharide synthesis. Reagents and conditions: (**a**) IBr/AgOTf, CH_2_Cl_2_/MeCN, −45 °C to rt, on, 70%; (**b**) (i) H_2_, Pd(OH)_2_, MeOH, rt, 2 days; (ii) NaOMe, MeOH, rt, 16 h; (iii) H_2_O, NaOMe, rt, 24 h; 94% (over three steps).

The synthesis of the NeuGc GM3 trisaccharide was carried out according to the scheme in Fig. 1. Initial glycosylation attempts between benzoyl protected 3′,4′-diol lactose acceptor **2**^31^ and donor **1**^32^ using IBr/AgOTf^33^ as promoter gave no sialylation product, only elimination of the Neu5Gc donor. However, using the same reaction conditions and donor, but changing the acceptor to the more active benzylated lactoside **3**^34^ efficiently afforded the α-linked sialylation product **4** in 70% yield. De-protection of **4** through catalytic hydrogenolysis followed by Zemplen deacylation and saponification afforded the target structure **5** in 94% yield.

### Design of scFv variants

**Figure 2:**
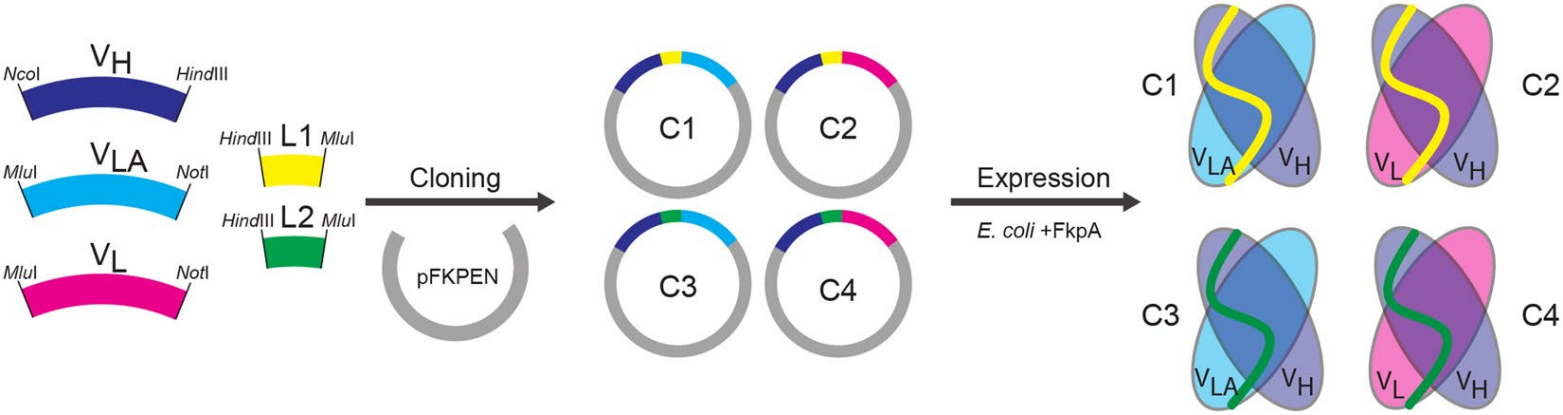
Design, cloning and expression of 14F7 derived scFv constructs C1-C4. Four scFv constructs were cloned into the periplasmic expression vector pFKPEN^35^, which constitutively expresses the periplasmic chaperone FkpA offering improved folding assistance of heterologous proteins.

Four different scFv variants of the original murine 14F7 were designed (Fig. 2 and Supplementary Data S1). In addition to the original 14F7 light chain, we used an alternative light chain variable region (V_LA_) based on previous phage display light chain shuffling experiments (clone 3Fm)^28^. Furthermore, we designed two linkers connecting V_H_ and V_L_ termed L1 and L2. L1 (*N-*KLSGSASAPKLEEGEFSEARV-*C*) was adapted from an established vector system for expression of scFvs in *Escherichia coli*^35^, while L2 (*N*-KLAPQAKSSGSGSESKVDARV-*C*) consists of an extended version of the linker used in the prior light chain shuffling screening^28^. The residues from the prior version are underlined. Matching V_H_ with either V_L_ or V_LA_ and linkers L1 or L2, gave rise to four different 14F7-derived scFv constructs named C1 to C4 (Fig. 2).

### Periplasmic expression and purification of 14F7-derived scFvs C1 to C4

**Figure 3:**
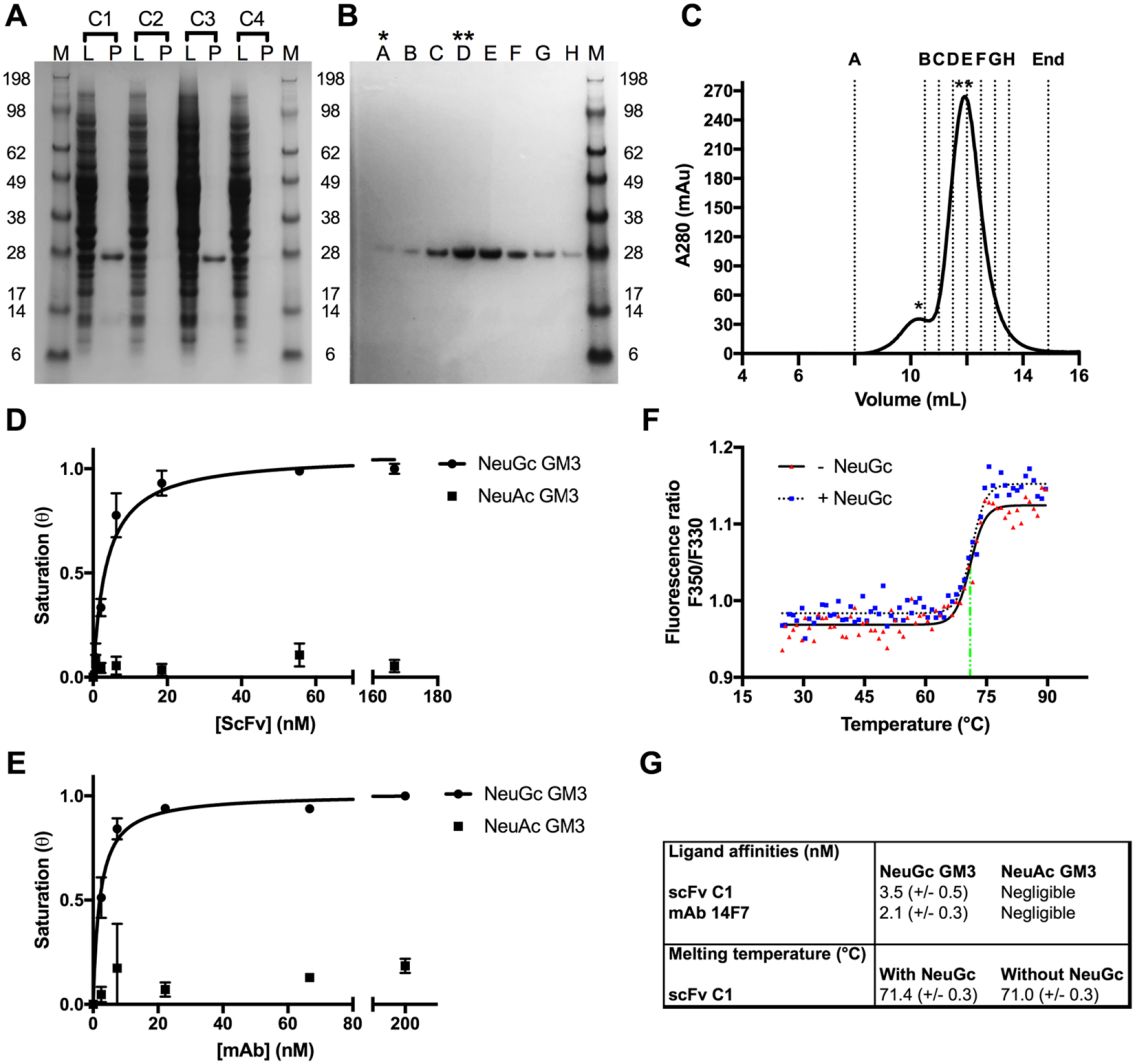
Production and characterisation of 14F7-derived scFvs and comparison to 14F7 mAb. (**A**) SDS-PAGE of periplasmic lysate (L) and purified protein (P) for scFv constructs C1-C4. (**B**,**C**) Representative SDS-PAGE (**B**) and size-exclusion chromatogram (**C**) for C1. Stars mark a minor fraction of dimerised protein (*) and the major fraction of monomeric scFv (**), respectively. (**D**,**E**) ELISA data fitted with a one-binding site model for 14F7-derived scFv C1 (**D**) and 14F7 mAb (**E**), respectively. Both 14F7 formats bind strongly to NeuGc GM3, but not to NeuAc GM3. (**F**) Thermostability of scFv C1 in the absence and presence of the NeuGc trisaccharide. (**G**) Summary of ligand affinities and melting temperatures.

The codon-optimised scFv 14F7 variants were cloned into an engineered pFPKEN vector that had a *pelB* leader sequence for translocation to the periplasm following translation. Two of the four constructs showed weak expression (C2 and C4), while the other two (C1 and C3) gave good yields (5 mg/L culture and 3 mg/L culture, respectively) (Fig. 3A). Both well-expressing constructs contain the alternative light chain 3Fm, in keeping with the earlier light chain shuffling experiments by Rojas *et al*.^28^. The highest quality protein was obtained by periplasmic extraction using the described buffer containing sucrose and lysozyme. Isolation and purification of the protein from whole cell lysate was also attempted and gave higher yields, but the scFvs appeared partly degraded based on SDS-PAGE analysis (Supplementary Data S2). Affinity purification using sepharose-coupled protein L proceeded immediately after isolation from the periplasmic fraction. Protein L binds to the majority of correctly folded antibody Vκ domains, and therefore offers a highly selective purification step for antibodies and fragments thereof with this domain^36^. During the initial purification, the scFv constructs were prone to proteolysis. Speed of work, keeping all solutions on ice and the presence of protease inhibitors were crucial to obtaining non-degraded protein, with an inhibitor cocktail showing the best effect. The 14F7 scFv fragments bound firmly to the protein L resin and eluted in the later fractions. In a second step, the protein was further purified using SEC, eluting in two peaks. The main fraction contained monomeric scFv (Fig. 3B,C). Repeating SEC using the monomeric fraction did not indicate further dimerization (not shown), therefore the protein was judged to be sufficiently stable and mono-disperse to allow down-stream characterisation and crystallisation. Storage of the scFv at −80 °C was possible without losing activity provided that the aliquoted protein samples were flash-frozen in liquid nitrogen. Based on the expression yields and purification efficiency, the scFv C1 construct was selected for detailed characterisation and crystallisation trials. This construct contains the alternative variable light chain V_LA_ and the L1 linker adapted from an established vector system for expression of single-chain T-cell receptors (scTCRs) and scFvs in *E. coli*^35,37^, which was not previously used for 14F7-related work.

### Stability, affinity and specificity of scFv C1

In order to assess the binding activity and selectivity of the designed 14F7 scFvs, the C1 representative was subjected to ganglioside-binding analysis using ELISA. NeuGc GM3 and NeuAc GM3 were immobilised on the plate and incubated with scFv C1 or control 14F7 mAb. Both antibody formats could clearly distinguish between the two gangliosides. They showed strong binding to the *N*-glycolyl variant and negligible binding to NeuAc GM3 (Fig. 3D,E). Fitting of the ELISA data yielded a *K*_D,app_ of 4 nM for scFv binding to NeuGc GM3 (Fig. 3D), compared to an apparent *K*_D,app_ of 2 nM for the mAb (Fig. 3E), in line with earlier experiments^28^. The thermostability of the scFv construct was estimated from a tryptophan fluorescence scan at increasing temperatures. A single clear transition occurred around 71.4 °C indicating the melting point of the protein. No significant difference was found in the presence of the NeuGc GM3 trisaccharide **5** (Fig. 3F). Both ELISA and thermostability measurements were carried out in triplicates. The data are summarised in Fig. 3G.

### Crystal structure of scFv C1

**Figure 4:**
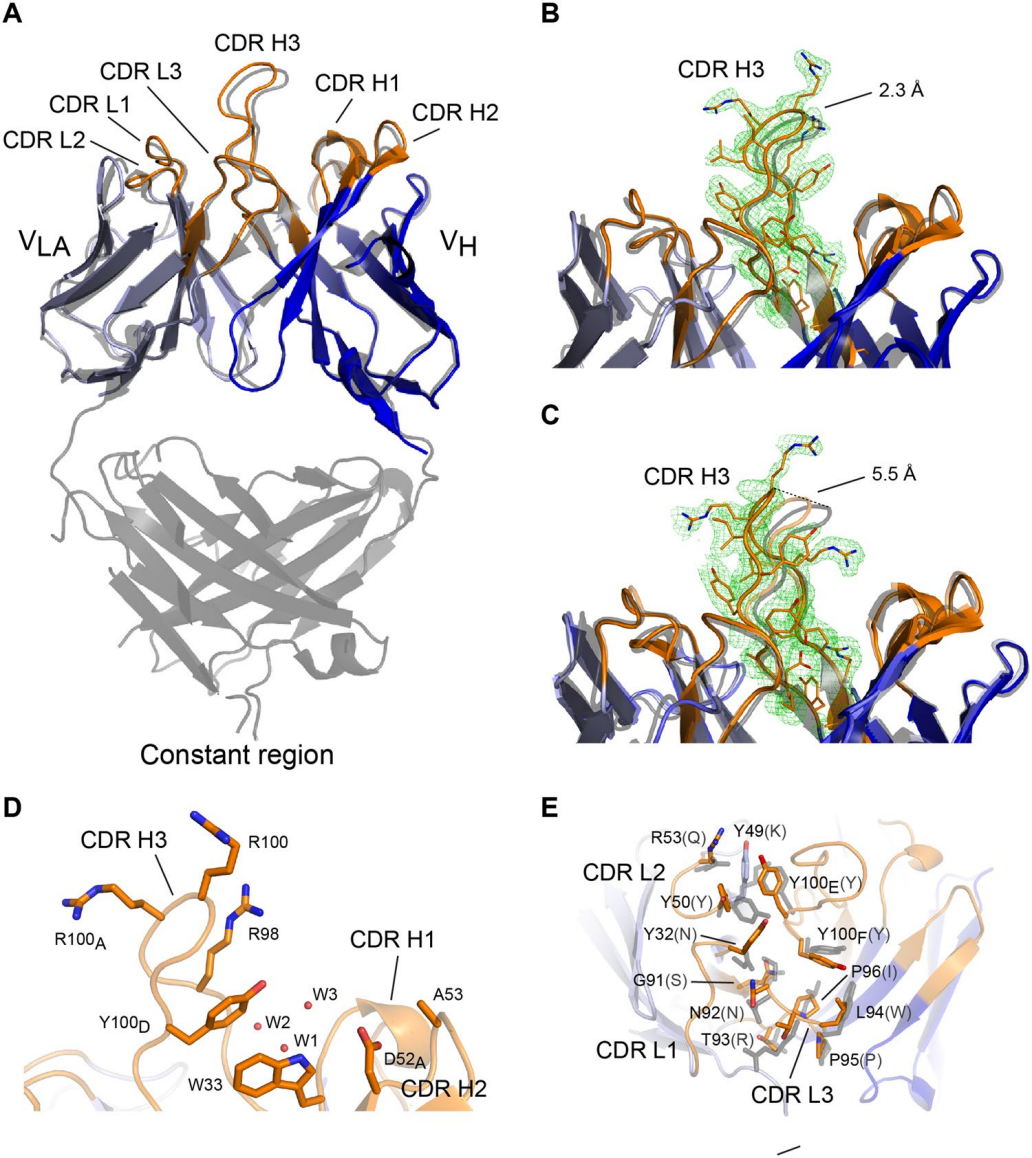
Structural comparison of scFv C1 to the 14F7 Fab. (**A**) Superimposition of scFv C1 heavy (dark blue) and light chains (light blue) on the equivalent domains of the 14F7 Fab (grey). CDR loops are coloured orange. Note that the 14F7 Fab contains V_L_ while scFv C1 contains V_LA_. Overall similarity is high, with largest differences at CDR H3 (shown here: scFv C1 chain A). (**B**,**C**) Close-up views of CDR H3. Comparison of scFv C1 molecules M1 and M2 with Fab CDR H3 (grey). (**B**) Shows the CDR H3 loop (orange) of M1, (**C**) shows the corresponding region of M2 compared to M1 (transparent orange) and Fab (grey). Difference (*m****F***_**o**_-*D****F***_**c**_) OMIT electron density maps (after simulated annealing) of the CDR H3 loop of M1 (**B**) and M2 (**C**) contoured at 2σ show that the loops are fully traceable. Main chain distances at the top of CDR H3 are marked with dashed lines. (**D**) Key residues of the CDR H3 loop targeted for mutation^30^. Three water molecules (W) are found in the suspected binding pocket in the best-defined scFv structures M1 and M2 (shown here: M1) (**E**) Interaction between V_LA_ and V_H_ with selected residues shown in stick representation (original V_L_ identity in grey parentheses). CDR L1-3 interact with V_H_ residues Tyr100_E_ and Tyr100_F_ (Kabat numbering^64^) and may therefore indirectly play a role in antigen recognition.

The crystal structure of scFv C1 was determined to 2.2 Å resolution. Despite the different light chains, it is structurally highly similar to the 14F7 Fab structure determined earlier to 2.6 Å resolution (PDB ID: 1RIH)^29^, with an all-atom r.m.s.d. value of 0.5 Å. The largest difference between the two structures is found in the CDR regions, notably in the important CDR H3 loop, which is subject to a small backbone shift (Fig. 4A). Based on mutation analysis and computational modelling, this loop was predicted to play an important role in NeuGc recognition^29^, which has recently been confirmed by phage display studies^30^. This work showed for example that CDR H3 residue Arg98 is essential for retaining binding affinity and cannot be substituted by any other amino acid without losing activity. While the exact position of Arg98 is governed by the shift in the backbone of the CDR H3 loop, the position in the scFv is comparable to that in the Fab. In the scFv C1 structure, four molecules (M1 to M4, see Supplementary Data S3) are present in the crystal’s asymmetric unit, which allows for an independent structural comparison of the CDR H3 loop. The loop cannot be fully traced in the electron density of two of these molecules (M3 and M4), which likely reflects some degrees of flexibility. In molecules M1 and M2 (Fig. 4B,C), however, the CDR H3 loops are well defined. In both cases the CDR H3 loop is engaged in different crystal contacts (see Supplementary Data S3). The CDR H3 conformation of M1 resembles that of the 14F7 Fab more closely than that of M2, with main chain distances at the loop tip of 2.3 Å and 5.5 Å, respectively (Fig. 4B,C, dashed lines). Another region that showed disorder in the crystal structure is the L1 linker region, which was not traceable in any of the four molecules, suggesting increased mobility compared to the rest of the molecule.

A second residue critical for NeuGc recognition is Trp33 in CDR H1^30^ (Fig. 4D). This residue is positioned exactly as in the 14F7 Fab. At the higher resolution of the current analysis, the structure reveals an extensive hydrogen bonding network in the suspected hydrophilic binding pocket^29^.

The differences in light chains between scFv C1 (V_LA_) and 14F7 mAb and Fab (V_L_) call for a detailed comparison. The sequence identity between the two light chains is 61%. A sequence alignment and stereo image of the scFv, with V_L_-V_LA_ differences mapped, can be found in Supplementary Data S4. We focused in particular on the interactions between the CDR regions of V_H_ and V_L_ or V_LA_. With 874 Å^2^ versus 828 Å^2^, the interface between V_LA_ and V_H_ in the scFv is somewhat larger than between V_L_ and V_H_ in the Fab, as calculated using PDBsum^38^. The interface is largely hydrophobic and quite tightly packed, featuring a number of aromatic residues involved in stacking interactions and hydrogen bonds. Especially important is the interaction with V_H_ CDR H3 (Fig. 4E). It is therefore plausible to assume that V_L_/V_LA_ indirectly contribute importantly to NeuGc GM3 binding, by stabilizing the characteristic long CDR H3 loop, configuring it for antigen recognition. Such stabilisation may be mediated directly, *e.g*. by π-π interactions of CDR H3 Tyr100_E_ with V_LA_ residues Tyr49 and CDR L2 Tyr50 from V_LA_ (Fig. 4E), as well as indirectly through a water-mediated hydrogen bonding network close to the interface with the scFv framework (Fig. 4D).

## Discussion

There is a high interest in antibody fragments that fully retain the antigen-binding capacity due to their application as building blocks in advanced immuno-therapeutics^39–41^. Several scFvs for therapeutic use are in development, however, none have entered the market. ScFvs have the advantage of rapid delivery and penetration of tumour cells compared to mAb molecules^42,43^. Their unique specificity renders them a versatile delivery vehicle for diagnosis, radio-immunotherapy and gene therapy, especially if fused to drugs or effector molecules^44^. Furthermore, the smaller scFv (28 kDa) is likely more crystallisable than the larger Fab or full mAb molecules. This work can therefore provide important structural information to guide further scFv development.

The production of scFvs, in the amounts and quality sufficient for structural determination, requires an efficient expression system that allows successful formation and maintenance of disulphide bonds crucial for structural integrity. The periplasmic chaperone FkpA enhances expression yields by facilitating folding and reducing protein degradation^45,46^. Here, we focused on the scFv derived from the 14F7 mAb, an anti-tumour antibody against NeuGc GM3 that has significant clinical potential^15,27^. The 14F7 Fab was crystallised more than a decade ago^29^, however, with very poor reproducibility; and in particular, there is still no crystal structure of the antigen complex. In this work, we therefore followed two aims: to provide good starting conditions to finally solve the structure of the 14F7 antigen complex and to more generally explore strategies to produce well-expressing scFvs.

In total four different constructs were produced and evaluated, based on two different variable light chains (V_L_ and V_LA_) and two different linkers (L1 and L2) (Fig. 2). Only two of these constructs, C1 and C3, expressed in adequate yields in XL1-Blue *E. coli* cells. Remarkably both C1 and C3 contain the alternative light chain variable region (V_LA_) previously selected from a phage-displayed light chain shuffling library^28^ based on the ability to pair with 14F7 V_H_ and allow the secretion of the corresponding scFv to the bacterial periplasm. Thus the need of replacing the original 14F7 V_L_ to achieve successful bacterial expression was confirmed in the context of a different vector/host strain system, implying that V_L_ somehow impairs the secretion process of the antibody fragments, whereas the sequence of the linker region appears to be of less importance in terms of expression levels. However, the linker may still affect stability as indicated generally for scFvs in computational studies^47^. In this work, two different linkers were used, both of which are slightly longer than those of the previous constructs by Rojas *et al*. and designed based on experiences by Løset and colleagues from expression of scTCRs and scFvs in *E. coli*^35,37^. The linker region was disordered in the crystal structure of scFv C1. Mutagenesis of parts of the linker to residues conferring structural rigidity and reduced conformational plasticity, as well as exhibiting higher propensity towards the formation of secondary structure elements (*i.e*., α-helix or β-sheet) may stabilise this region more and reduce proneness to aggregation and/or unwanted proteolytic activity. In addition, this may also improve chances of crystallisation. Indeed, in another study, the L1 linker used herein was subject to a Leu to Pro mutation in the second position when channelled through thermostability engineering as part of a highly unstable scTCR^48^. We further note that the elongation of the linker from *N*-EKSSGSGSESKVD-*C*^28^ to *N*-KLAPQAKSSGSGSESKVDARV-*C* (as in L2) possibly releases a strain between the domains, allowing for the detailed structural characterisation presented here.

Apart from the construct design, the expression and purification conditions were important. For example, we found that periplasmic isolation of the protein was worthwhile, despite the loss in overall yields, due to the significantly improved scFv quality. The protein molecules were much less degraded (compare Fig. 3A and Supplementary Data S2). Additional benefits were achieved by working at 4 °C throughout and using a protease inhibitor cocktail rather than individual inhibitors. Several attempts to increase yields by induction with IPTG were found to be counterproductive and we therefore assume that the leaky basal expression is close to optimal at the levels of chaperone present.

The scFv C1 construct was tested for binding activity using ELISA and compared with the 14F7 mAb. The data clearly show that scFv C1, just as the 14F7 mAb, can discriminate strongly between NeuGc and NeuAc GM3, and barely binds the latter. This is important for potential clinical applications of the humanised antibody. While the ELISA experiment is a relatively crude method for determining the *K*_D_, it nevertheless indicates that the *K*_D_ of the scFv is comparable to the apparent *K*_D_ of the mAb with respect to NeuGc GM3 binding. The strong binding affinity, differentiation between NeuGc and NeuAc, and high thermostability make scFv C1 a very attractive molecule for drug delivery and immunotherapy. Surprisingly, no further increase in thermostability was observed when bound to the NeuGc GM3 trisaccharide **5**. This could indicate that the lipid part of the ganglioside contributes importantly to binding and the trisaccharide has limited binding affinity on its own. Alternatively, since its thermostability is already high, it is possible that **5** binds strongly to its target, but that the stabilizing effect of binding is nevertheless negligible in the already highly stable scFv.

Structural analysis of scFv C1 revealed that the important CDR H3 region adopts a similar conformation as in the 14F7 Fab, stabilised by tight interactions with the light chain CDRs. There is, however, some variation between the four different molecules in the crystal. For M1 and M2, the electron density was of sufficient quality to allow tracing of the chains. They adopt slightly different conformations, which at the tip diverge by up to 5.5 Å from the Fab structure (Fig. 4C). The different conformations may provide suitable alternative starting positions for *in silico* modelling of the antigen complex. An additional benefit of the scFv structure compared to the Fab is its higher resolution. While the conformation responsible for recognizing the NeuGc GM3 antigen may only be revealed experimentally, the dynamic nature of the CDR H3 may be important for the actual recognition mechanism. After all, this long loop has to be inserted quite deeply into the biological membrane, as discussed in more detail below.

Phage display-based directed evolution experiments showed that while most substitutions in CDR H3 abolished binding to NeuGc GM3, mutation of Trp33 to Phe, Tyr or Gln rescued binding affinity^30^. Furthermore, Trp33Gln produced moderate cross-reactivity to NeuAc GM3. The position of Trp33 in the scFv is identical to that of the Fab, but at the higher resolution, additional water molecules were identified. These water molecules contribute to a hydrogen bonding network that may be important for ligand recognition (Fig. 4D). Furthermore, the maintenance of aromaticity without hydrogen bonding capability resulting from the Trp33Phe mutation^30^ suggests that a π interaction at this position may be essential to NeuGc specificity. The other rescuing variant, Trp33Gln, suggests that Gln may give rise to an alternative hydrogen bonding network that still supports trisaccharide binding, but compromising specificity.

Previous work indicated that it is mainly, if not exclusively, the prominent CDR H3 loop that recognises the antigen^28–30^. However, a comparison of the V_L_-V_H_ interface of the different variable light chains of the Fab and scFv C1 structures (V_L_ and V_LA_, respectively) reveals important interactions that may be indirectly linked to antigen affinity (Fig. 4E). These include the π-π interactions of Tyr100_E_ found in CDR H3 with residues of the V_L_ and V_LA_ CDR L1. Interestingly, computational studies indicate that destabilisation of the V_L_ CDRs may lead to an increased stability of V_H_ CDRs and *vice versa*^47^, thus suggesting that more distal, indirect effects on protein dynamics by mutations in CDR regions could affect ligand binding.

One intriguing aspect of the small NeuGc GM3 glycan head group is that it would bring the scFv in close proximity to the plasma membrane, thus logically implying a potential interaction with lipids surrounding the ganglioside. In fact, molecular dynamics simulations have suggested that GM3 is deeply embedded in the cell membrane and that only the terminal saccharides are exposed^49,50^. Moreover, the interaction of gangliosides with cholesterol in lipid rafts appears to bend the glycolipid head group into a conformation almost parallel to the membrane^51,52^. Recently, internalizing scFvs have been described and even selected for by phage display^53,54^. Selecting for this trait in conjunction with recognition of NeuGc GM3 may provide a very potent delivery molecule for future antibody-drug conjugate development. The stable production and structure determination of this active scFv, which retains high binding affinity and stability, is a good starting point for the structural characterisation of the antigen complex. Such a structure would finally reveal the molecular details of NeuGc GM3 selectivity and moreover provide the basis for scFv – membrane interactions, contributing valuable information to the development of 14F7-derived therapeutics. Last but not least, the work described here provides valuable new insight and general guidelines for the production of well-expressing scFvs.

## Methods

### Synthesis of NeuGc trisaccharide

#### Methyl (4,7,8,9-tetra-*O*-acetyl-5-benzyloxyacetamido-3,5-di-deoxy-α-d-*glycero*-d-*galacto*-2-nonulopyranosyl)onate-(2 → 3)-2,6-di-*O*-benzyl-β-d-galactopyranosyl-(1 → 4)-1,2,3,6-tetra-*O*-benzyl-β-d-glucopyranoside (4)

A mixture of acceptor **3** (0.200 g, 0.226 mmol), donor **1** (0.170 g, 0.272 mmol) and molecular sieves (3 Å) in dry CH_2_Cl_2_ (3 mL) was stirred for 20 min under a nitrogen atmosphere, when a solution of AgOTf (0.102 g, 0.678 mmol) in dry MeCN (4.5 mL) was added. After 15 min of stirring at room temperature, the reaction mixture was cooled to −45 °C. Thereafter a solution of IBr (1.0 M in CH_2_Cl_2_, 0.45 mL, 0.452 mmol) was added dropwise. The reaction was stirred and gradually allowed to warm to −30 °C, then stirred for a total reaction time of 3 h. Diisopropylamine was added to neutralise the reaction and the stirring was continued for another 20 min. The mixture was filtered through a pad of Celite, and the filtrate concentrated *in vacuo*. The residue was purified by flash column chromatography on silica gel (toluene/MeCN, 3:1) to give **4** (0.231 g, 70%) as a white solid. [**α**]_D_ + 2.3 (c 1.0, CHCl_3_). The correct product was verified by NMR and mass spectrometry. ^1^H NMR (500 MHz, CDCl_3_) chemical shifts can be found in Supplementary Data S5.

#### 3,5-Di-deoxy-5-glycoylamido-α-d-glycero-d-galacto-2-nonulopyranosylonic acid-(2 → 3)-β-d-galactopyranosyl-(1 → 4)-d-glucopyranose (5)

10% w Pd(OH)2 (75 mg) was added to **4** (0.100 g, 0.068 mmol) in MeOH (5 mL). The reaction mixture was stirred under a hydrogen atmosphere (using a balloon) at room temperature for 2 days to complete conversion according to TLC. The reaction was filtered through PTFE frits (3 frits stacked on top of each: 20 μm, 10 μm, 5 μm) and rinsed with MeOH. The filtrate was concentrated *in vacuo* to give a crude de-benzylated compound as a white solid. Freshly prepared sodium methoxide was added to a solution of the crude product dissolved in dry methanol (3 mL) under a nitrogen atmosphere. The mixture was stirred at room temperature until complete conversion (16 h) according to TLC. Then water (0.5 mL) was added to the reaction mixture and additional sodium methoxide was added to reach a pH of 12. The reaction was stirred at room temperature for 24 h and then neutralised by the addition of Dowex H^+^ ion exchange resin. The resin was filtered off and washed with methanol and water. The filtrate was concentrated to a crude product, followed by freeze drying to afford an anomeric mixture (α:β, 6:11) of compound **5** (0.042 g, 94%) as a light white solid. The correct product was verified by NMR and mass spectrometry. ^1^H NMR (500 MHz, CDCl_3_) chemical shifts can be found in Supplementary Data S5.

### Cloning of scFv variants

For efficient expression in *E. coli*, codon-optimised complete scFv cassettes encoding either the 14F7 V_L_ and V_H_ domains (GenBank accession no.: AY331717 and AY331718), or a variant containing the alternative 3Fm V_LA_ domain^28^, were synthesised (Life technology). Four scFv constructs were designed, named C1-C4, which combine different variable light chains and linkers (Fig. 2). The linkers were cloned to bridge the V_H_ and V_L_ inserts using 5′*Hind*III and 3′*Mlu*I sites with matching sites on the 3′ end of V_H_ and 5′ end of V_L_ to complete the inserts. The full inserts of 14F7 scFvs were then sub-cloned into the bacterial expression vector pFKPEN using 5′ *Nco*I and 3′ *Not*I restriction sites, thus appending a pelB leader for periplasmic expression. In addition, the vector encodes a constitutively expressed periplasmic foldase FkpA^35^ as chaperone.

### Expression and purification of scFv constructs

14F7-derived scFvs C1-4 were expressed in XL1-Blue *E. coli* cells. Transformed cells were grown overnight in 2x YT medium supplemented with 2% glucose and 100 μg/mL ampicillin (2x YT_GA_). Other media (Lysogeny Broth and Terrific Broth) were also tested, and Terrific Broth gave almost as good yields as YT medium. Overnight cultures were used to inoculate larger volumes and were allowed to grow at 37 °C at 125 rpm until OD_600_ reached 0.6–0.8. The cells were pelleted at 4000 × *g* for 40 minutes at 4 °C. Pellets were re-suspended in equal volumes of 2x YT without glucose (2x YT_A_) to allow protein expression at 30 °C overnight. No IPTG was added to induce expression, after tests revealed that the application of IPTG leads to lower protein quality. Cells were harvested the following morning by centrifugation at 4000 × *g* at 4 °C. Periplasmic extracts were prepared by re-suspending the pellets in extraction buffer (50 mM Tris-HCl pH 7.5, 20% sucrose, 1 mM EDTA, 80 μg/μL lysozyme, 80 μg/μL DNase, cOmplete protease inhibitor (Sigma)), and yielded approximately 5 ml per gram cell pellet. The solutions were stirred for one hour on ice, before the soluble fractions were isolated by centrifugation at 18000 rpm for 30 min at 4 °C. The periplasmic extracts were loaded on a 1 mL protein L column (Pierce), washed with PBS, and the captured proteins were eluted with 0.1 M glycine, pH 2.5. Following elution, fractions were immediately neutralised with 1 M Tris-HCl pH 7.5. The proteins were further purified by size exclusion chromatography (SEC). SEC of scFv C1 used in subsequent crystallisation and diffraction experiments was done in 20 mM Tris-HCl pH 7.5 and 150 mM NaCl. SEC of scFv used for downstream characterisation experiments (ELISA, thermostability), was performed in PBS. Protein concentrations were determined (Implen NanoPhotometer) using extinction coefficients calculated from the protein sequences by ProtParam^55^ (Supplementary Data S1). Protein integrity was assessed by reducing SDS-PAGE and visualised by Coomassie staining.

### Binding assays using ELISA

NeuGc GM3 was obtained from horse erythrocytes using a modification of the Folch method^56^. NeuAc GM3 was purchased (Avanti Polar Lipids). Both gangliosides were solubilised in methanol. All steps of the protocol were performed at room temperature. The wells of a Nunc-Immuno 96 MicroWell PolySorp solid plate (Sigma) were coated with 100 μL ganglioside solution at 10 μg/mL and left to dry overnight. The next morning the wells were washed three times with 100 μl PBS_T_ per well (1x PBS containing 0.1% Tween 20) before the addition of 200 μl of PBS_TB_ (PBS_T_ containing 2% BSA). The blocking solution was left for one hour followed by a 3x PBS_T_-wash. The scFv C1 stock solution was prepared in PBS_TB_ at concentrations: 2.10, 6.20, 18.5, 55.6, 167 and 500 nM. The 14F7 mAb was prepared in PBS_TB_ at concentration: 3.70, 11.1, 33.3, 100 and 300 nM. The mAb functions as a positive control, and two negative controls were also included: PBS_TB_ (no protein added) to map background absorption and an unrelated scFv to control for unspecific binding. All samples were set up in triplicates, using 100 μl protein solution per well to ensure reproducibility. The samples were incubated for one hour, followed by a 3x PBS_T_-wash. The targetbound scFv or mAb were detected using protein L coupled horseradish peroxidase (HRP, Genscript), diluted in 1x PBS (1:2000). 100 μl were added to each well and incubated for one hour. Subsequently, the plates were washed four times with PBS_T_, followed by the addition of 100 μl substrate 3,3′,5,5′-tetramethylbenzidine (TMB). The developed signal was stopped by the addition of 100 μl 1 M HCl to each well and absorbance measured at 450 nm by a Multimode microplate reader (Thermo Scientific). The ELISA data were background subtracted and normalised to the data point of the highest protein concentration assuming saturation at this point ($\theta$ = 1). A simple one-binding-site model, $\theta =\frac{[{protein}]}{[{protein}]+{K}_{D,{app}}}$, where [*protein*] is the actual concentration of either mAb or scFv. *K*_D,app_ is the apparent dissociation constant of the system. Due to the potentially bivalent interaction of the mAb (two binding sites), the value does not represent a true *K*_D_ for these.

### Assessment of scFv thermostability

The thermostability of scFv C1 was determined by recording the tryptophan fluorescence signal while heating the protein sample. Measurements were done in a JASCO-8500 fluorimeter equipped with a Peltier heating unit. A protein concentration of 2 μM was used. The sample was placed in a 200 μL cuvette, excited at 295 nm and stirred with a magnetic bead at 200 rpm during the entire measurement. The emission at 350 nm was recorded with the photo multiplier set to high sensitivity. The sample was exposed to a temperature gradient from 25 °C to 90 °C at ΔT = 1 °C/min. Melting point determination was done in the presence and absence of 20 μM NeuGc GM3 trisaccharide **5**. The tryptophan fluorescence intensity ratio at between 350 nm and 330 nm (F350/F330) was calculated. This ratio represents a shift from the folded to the denatured and thus solvent exposed tryptophan residues. A Boltzmann distribution was fitted to the data in order to determine the melting temperature T_m_ at the inflection point using Graphpad Prism 7.

### Crystallisation

Purified scFv was concentrated to 16 mg/mL and crystallised using the Structure screen (Molecular Dimensions). Single crystals were obtained from condition G4 (0.1 M sodium HEPES pH 7.0, 20% w/v PEG 10 K) and flash-cooled in liquid nitrogen in the presence of a range of cryo-protectants. Although diffraction experiments showed reflections to approximately 2 Å resolution, the acquired data could not be used for further structure determination. Instead we used the G4 condition to microseed crystals into the cryo-protected Morpheus screen (Molecular Dimensions). Small plate-like crystals appeared and were mounted in a litho-loop directly from the D12 condition (12.5% w/v PEG 1000, 12.5% w/v PEG 3350, 12.5% v/v MPD, 0.02 M 1,6-hexandiol, 0.02 M 1-butanol, 0.02 M(RS)-1,2-propanediol, 0.02 M 2-propanol, 0.02 M 1,4-butanediol, 0.02 M 1,3-propanediol, 0.1 M Bicine/Tris base pH 8.5). The crystals were flash-cooled in liquid nitrogen and stored for diffraction experiments.

### Data collection and structure determination

Diffraction data to 2.2 Å resolution were collected from a single crystal at the ID29 micro focus beam line at the European Synchrotron Radiation Facility (ESRF) Grenoble, France. The data were processed with the *EDNA* auto-processing procedure provided at the beam line^57^. The space group was determined to be *P*2_1_ and the data cut-off chosen based on the correlation coefficient (CC_1/2_) statistics in accordance with ref.^58^. We decided to use a conservative cut-off in CC_1/2_ of 0.80 after evaluation of map quality and refinement *R*-factors. The most probable number of molecules in the asymmetric unit was determined to be four from the Matthews coefficient^59^. The data were phased by molecular replacement using a homology model of scFv C1 based on PDB entry 3UMT, another scFv with 65% sequence identity to C1. Molecular replacement was done using *Phaser*^60^ from the *PHENIX* suite^61^. The homology model was prepared by *SWISS-MODEL*^62^. Based on the phased data, an initial experimental model was built using *phenix.autobuild*. The final model was obtained after several cycles of refinement (*phenix.refine*) and manual model building with *Coot*^63^. The model was numbered according to the Kabat system^64^. Data collection and refinement statistics are summarised in Table 1. Figures of the final experimental model were generated with *PyMol* (PyMol Molecular Graphics System, Version 1.8 Schrödinger, LLC). The final model was deposited in the Protein Data Bank (PDB) with accession code 6FFJ.

**Table 1:** X-ray crystallographic data collection and refinement statistics.

	scFv C1 (PDB ID 6FFJ)*
**Data collection**	
Beam line Wave length (Å) Space group	ID-29, ESRF 1.0332 *P*2_1_
Cell dimensions	
*a*, *b*, *c* (Å)	63.8 119.1 68.3
*α, β, γ* (°)	90 90.2 90
Solvent content (%) Resolution (Å)	51.0 46.7-2.15 (2.28-2.20)**
*R*_sym_ (%) *R*_*meas*_ (%)	6.4 (50.4) 13.7 (65.3)
*I*/σ(*I)*	12.5 (1.2)
Completeness (%)	95.2 (96.4)
Multiplicity CC 1/2 Wilson *B*-factor (Å^2^)	2.5 (2.5) 0.99 (0.80) 37.8
**Refinement**	
Resolution (Å)	46.7- 2.20
No. unique reflections	48225 (4971)
No. reflections in test set *R*-work/*R*-free	2495 (260) 0.199/0.243
No. atoms	
Protein	7202
Water	254
*B*-factors (Å^2^)	
Protein	44.0
Water	41.2
R.m.s. deviations	
Bond lengths (Å)	0.010
Bond angles (°)	1.05
Ramachandran plot Favoured (%) Outliers (%)	97.3 0.1

*Data collected on a single crystal.

**Values in parentheses are for highest-resolution shell.

Other datasets generated during and/or analysed during the current study are available from the corresponding authors upon reasonable request.

## Electronic supplementary material

Supplementary data
