# Supplementary material for: Crystal structure of an L chain optimised 14F7 anti-ganglioside Fv suggests a unique tumour-specificity through an unusual H-chain CDR3 architecture
Source: Sci Rep. 2018 Jul 18;8:10836. doi: 10.1038/s41598-018-28918-5 (PMC6052152; doi:10.1038/s41598-018-28918-5)
Supplement: Supplementary file 1 — Supplementary data [file 41598_2018_28918_MOESM1_ESM.pdf]

Supplementary Material

Crystal structure of an L chain optimised 14F7 anti-ganglioside Fv suggests a unique tumour-specificity through an unusual H-chain CDR3 architecture

Kaare Bjerregaard-Andersen[1*#], Hedda Johannesen[1#], Noha Abdel-Rahman[1,2], Julie Elisabeth Heggelund[1§], Helene Mykland Hoås[1], Fana Abraha[3], Paula A. Bousquet[1], Lene Støkken Høydahl[4], Daniel Burschowsky[1†], Gertrudis Rojas[5], Stefan Oscarson[3], Geir Åge Løset[4,6,7*], Ute Krengel[1*]

[1] Department of Chemistry, University of Oslo, NO-0315 Oslo, Norway
[2] Department of Biochemistry, Faculty of Pharmacy, Mansoura University, Mansoura 35516, Egypt
[3] School of Chemistry, University College Dublin, Belfield, Dublin 4, Ireland
[4] Centre for Immune Regulation and Department of Immunology, University of Oslo and Oslo University Hospital, N-0372 Oslo, Norway.
[5] Center of Molecular Immunology, Calle 216 esq 15, Atabey, Playa, La Habana CP 11300, Cuba
[6] Department of Biosciences, University of Oslo, NO-0316 Oslo, Norway
[7] Nextera AS, Oslo, Norway

Present addresses:
[§] School of Biomedical Sciences, University of Leeds, Leeds, LS2 9JT, UK
[†] Leicester Institute of Structural and Chemical Biology, University of Leicester, Leicester, LE1 7HB, UK

[#]Authors contributed equally
[*]Correspondence: Kaare Bjerregaard-Andersen (kaarebj@kjemi.uio.no), Geir Åge Løset (g.a.loset@ibv.uio.no) and Ute Krengel (ute.krengel@kjemi.uio.no).

Supplementary Data

Supplementary Data S1:

Translated protein sequences and calculated extinction coefficients for constructs C1-C4. Format: V_H – linker - V_L. Linker sequence is underlined.

C1:

QVQLQQSGAELAKPGASMKMSCRASGYSFTSYWIHWLKQRPDQGLEWIGYIDPATAY
TESNQKFKDKAILTADRSSNTAFMYLNSLTSEDSAVYYCARESPRLRRGIYYYAMDYWGQ
GTTVTVSSKLSGSASAPKLEEGEFSEARVDIQMTQTPSSLSASLGDRVTISCRASQDISN
YLNWYQQKPDGTVKLLIYYTSRLHSGVPSRFSGSGSGTDYSLTISNLEQEDIATYFCQQG
NTLPPTFGAGTKLELK

Ext. Coeff. (280 nm): 53080 M^{-1} cm^{-1}
Theoretical pI: 6.9

C2:

QVQLQQSGAELAKPGASMKMSCRASGYSFTSYWIHWLKQRPDQGLEWIGYIDPATAY
TESNQKFKDKAILTADRSSNTAFMYLNSLTSEDSAVYYCARESPRLRRGIYYYAMDYWGQ
GTTVTVSSKLSGSASAPKLEEGEFSEARVDLVLTQSPATLSVTPGDSVSFSCRASQSISN
NLHWYQQRTHESPRLLIKYASQSISGIPSRFSGSGSGTDFTLSISSVETEDFGMYFCQQS
NRWPLTFGAGTKLELK

Ext. Coeff. (280 nm): 54110 M^{-1} cm^{-1}
Theoretical pI: 7.8

C3:

QVQLQQSGAELAKPGASMKMSCRASGYSFTSYWIHWLKQRPDQGLEWIGYIDPATAY
TESNQKFKDKAILTADRSSNTAFMYLNSLTSEDSAVYYCARESPRLRRGIYYYAMDYWGQ
GTTVTVSSKLAPQAKSSGSGSESKVDARVDIQMTQTPSSLSASLGDRVTISCRASQDISN
YLNWYQQKPDGTVKLLIYYTSRLHSGVPSRFSGSGSGTDYSLTISNLEQEDIATYFCQQG
NTLPPTFGAGTKLELK

Ext. Coeff. (280 nm): 53080 M^{-1} cm^{-1}
Theoretical pI: 8.6

C4:

QVQLQQSGAELAKPGASMKMSCRASGYSFTSYWIHWLKQRPDQGLEWIGYIDPATAY
TESNQKFKDKAILTADRSSNTAFMYLNSLTSEDSAVYYCARESPRLRRGIYYYAMDYWGQ
GTTVTVSSKLAPQAKSSGSGSESKVDARVDLVLTQSPATLSVTPGDSVSFSCRASQSISN
NLHWYQQRTHESPRLLIKYASQSISGIPSRFSGSGSGTDFTLSISSVETEDFGMYFCQQS
NRWPLTFGAGTKLELK

Ext. Coeff. (280 nm): 54110 M^{-1} cm^{-1}
Theoretical pI: 8.8

Supplementary Data S2:

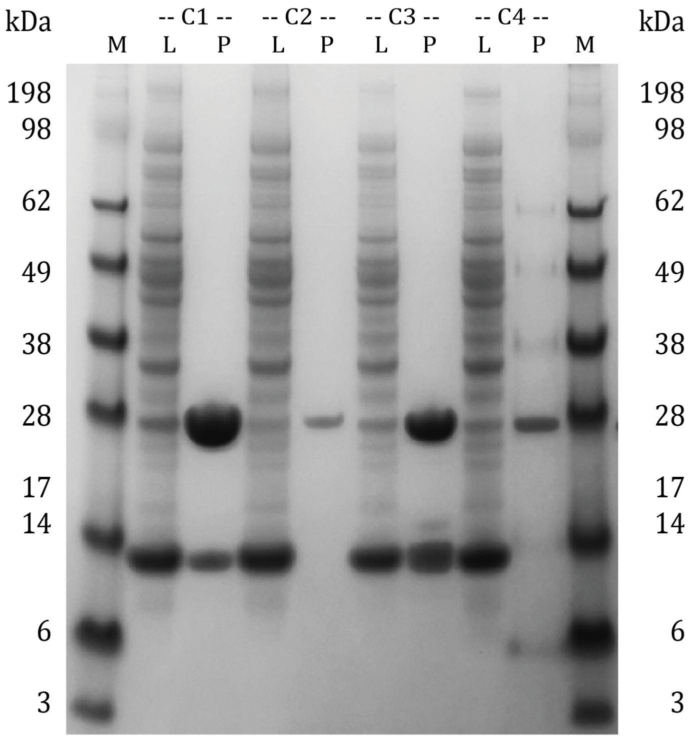

Purification of scFv C1-4 from whole cell lysate. Lanes with lysate (L) and purified scFv (P) are labelled. Marker (M) is shown for reference.

Supplementary Data S3:

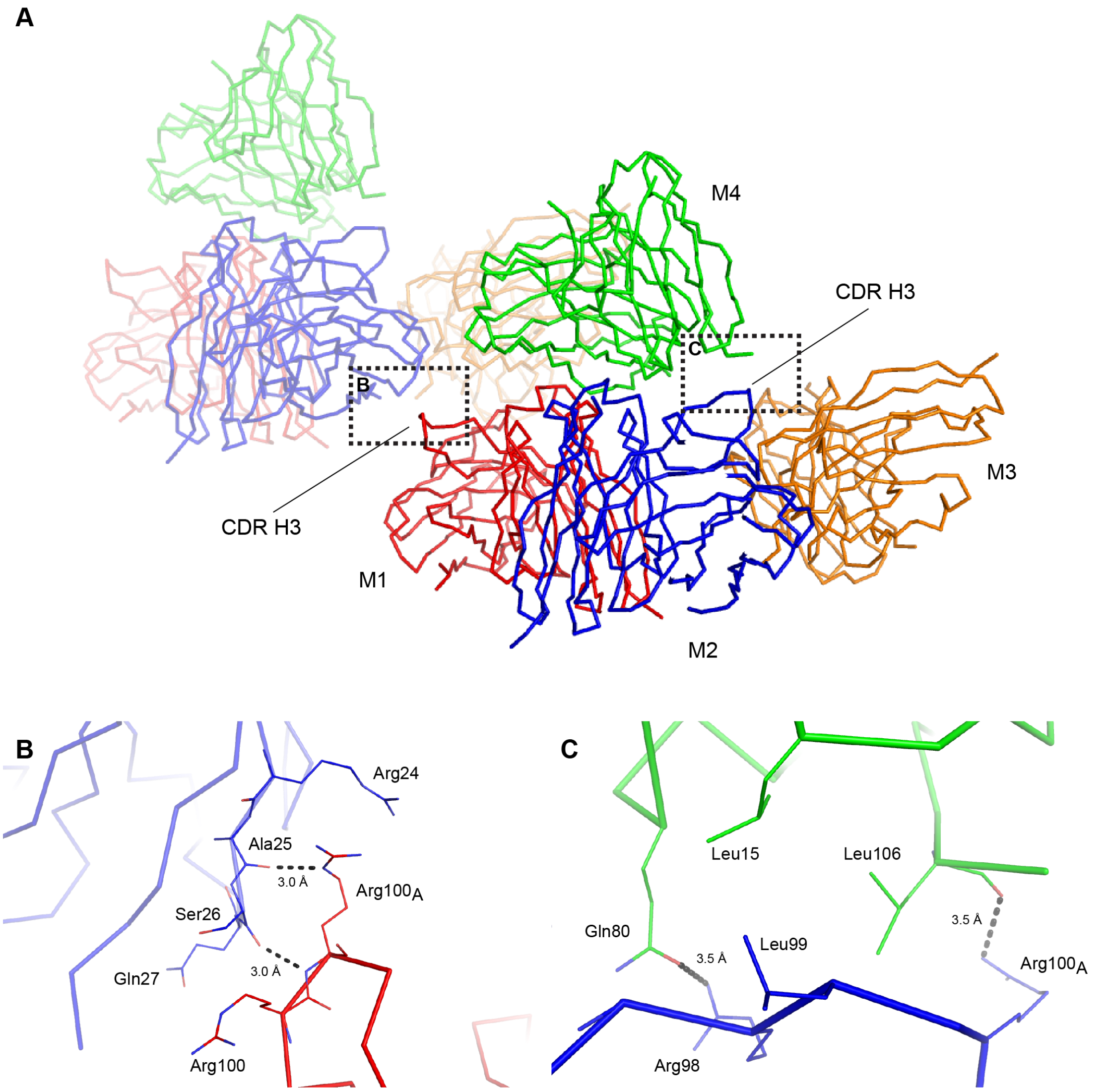

Crystal contacts of the CDR H3 loops. **(A)** Molecules M1 (red), M2 (blue), M3 (orange) and M4 (green) are depicted and symmetry-related molecules are displayed as transparent Cα traces with same colour coding. **(B)** The CDR H3 loop of M1 primarily interacts with M2. Arg100$_A$ interacts by hydrogen bonding with its side chain and main chain to Ala25 and Ser26 backbone carbonyls. Furthermore, there may be stacking interactions with Arg24. **(C)** The CDR H3 loop of M2 primarily interacts with M4. A motif of three leucine residues – Leu15, Leu99 and Leu106 – is central to the interaction. CDR H3 residues Arg98 and Arg100A are moreover close to residue Gln80 and the backbone carbonyl of Leu106, respectively, however, the distances (3.5 Å) are too long for efficient hydrogen bonding.

Supplementary Data S4:

A

```
VL   DLVLTQSPATLSVTPGDSVSFSCRASQSISNNLHWYQQRTHESPRLLIKYASQSISGIPSRFSG
     |. .||.|..|| . || |..|||||| ||| |.||||. . . .||| |.|.  ||.||||||
VLA  DIQMTQTPSSLSASLGDRVTISCRASQDISNYLNWYQQKPDGTVKLLIYYTSRLHSGVPSRFSG

VL   SGSGTDFTLSIISVETEDFGMYFCQQSNRWPLTFGAGTKLELK
     ||||||..|.| ..| ||.. |||||.|  | |||||||||||
VLA  SGSGTDYSLTISNLEQEDIATYFCQQGNTLPPTFGAGTKLELK
```

B

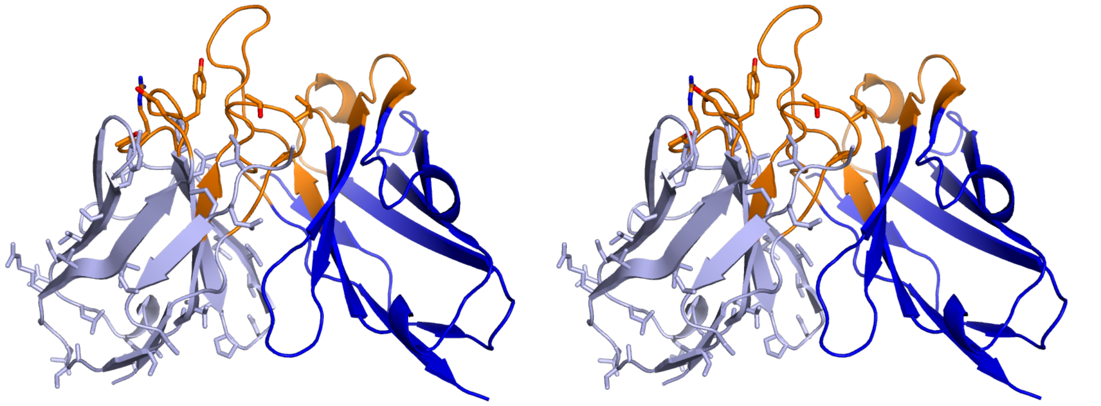

Comparison of scFv light chains. (**A**) Alignment of V_{LA} and V_L sequences. Identical residues are marked with lines, similar residues with dots. Percentage ID = 60.75. (**B**) Stereo image of scFv C1 structure (PDB ID: 6FFJ; this work). V_{LA} and V_H are shown in light and dark blue, respectively, CDRs are coloured orange. Residues differing between V_{LA} (3Fm variant, Rojas *et al.* 2004; present in C1) and the original V_L domain are shown as sticks.

Supplementary Data S5:

NMR chemical shifts and mass spectrometry data for products in the synthesis of NeuGc trisaccharide

Methyl (4,7,8,9-tetra-*O*-acetyl-5-benzyloxyacetamido-3,5-di-deoxy-α-D-*glycero*-D-*galacto*-2-nonulopyranosyl)onate-(2→3)-2,6-di-*O*-benzyl-β-D-galactopyranosyl-(1→4)-1,2,3,6-tetra-*O*-benzyl-β-D-glucopyranoside (4)

^{1}H NMR (500 MHz, $CDCl_3$). δ 7.40-7.20 (m, 35H, H-Ar), 6.30 (d, J = 10.3 Hz, 1H, NH), 5.44-5.41 (m, 1H, H-8III), 5.32-5.29 (m, 1H, H-7III), 4.99-4.84 (m, 4H, 3 x CH_{2a}-Bn, H-4II), 4.79-4.65 (m, 5H, 3 x CH_{2b}-Bn, CH_2-Bn), 4.63-4.33 (m, 8H, 3 x CH_2-Bn, H-1II, H-1I), 4.25 (dd, J = 12.6, 2.7 Hz, 1H, H-9aIII), 4.13 (q, J = 10.4 Hz, 1H, H-5III), 4.10-4.02 (m, 2H, H-3II, H-6III), 4.00-3.95 (m, 2H, H-4I, H-9bIII), 3.92-3.81 (m, 3H, CH_2CO, H-4II), 3.77 (s, 3H, OCH_3), 3.75-371 (m, 2H, H-6abI), 3.72-3.66 (m, 1H, H-6aII), 3.59-3.52 (m, 2H, H-3I, H-2II), 3.52-3.45 (m, 3H, H-6bII ,H-5II, H-2I), 3.37-3.33 (m, 1H, H-5I), 2.66 (d, 1H, OH), 2.55 (dd, J = 13.0, 4.6 Hz, 1H, H-3eqIII), 2.09 (s, 3H, COCH3), 2.03 (t, J = 12.8 Hz, 1H, H-3axIII), 1.98 (s, 3H, $COCH_3$), 1.96 (s, 3H, $COCH_3$), 1.88 (s, 3H, $COCH_3$); **^{13}C NMR** (125 MHz, $CDCl_3$) δ 170.7, 170.4, 170.1, 170.0 (4 x COCH3), 168.5 (CO2CH3), 139.3, 139.1, 138.8, 138.7, 138.6, 137.8, 136.9, 129.2, 128.8, 128.5, 128.4, 128.4, 128.4, 128.3, 128.3, 128.3, 128.2, 128.2, 128.0, 127.8, 127.7, 127.7, 127.6, 127.6, 127.5, 127.3 (42 x C-Ar), 102.6 (C-1I), 102.5 (C-1II), 98.5 (C-2III), 83.2 (C-3I), 82.1 (C-2I), 78.6 (C-2II), 76.6, 76.6 (H-4I, H-3II), 75.5 (CH_2-Bn), 75.3 (C-5I), 75.1, 75.1 (2 x CH_2-Bn), 73.7, 73.5, 73.2 (3 x CH_2-Bn), 72.7, 72.7 (C-6III, C-5II), 71.1 (CH_2-Bn), 69.3 (CH_2CO), 69.1 (C-4III), 68.7 (2C, C-6I, C-6II), 68.6 (C-8III), 68.1 (C-4II), 67.3 (C-7III), 62.4 (C-9III), 53.2 (OCH_3), 48.5 (C-5III), 36.8 (C-3III), 21.3, 20.9, 20.8, 20.7 (4 x $COCH_3$); **HRMS (ESI)**: m/z calcd for $C_{81}H_{91}NO_{24}$ $[M+Na]^+$, 1484.5829; found, 1484.5842.

3,5-Di-deoxy-5-glycoylamido-α-D-*glycero*-D-*galacto*-2-nonulopyranosylonic acid-(2→3)-β-D-galactopyranosyl-(1→4)-D-glucopyranose (5).

β-anomer ^{1}H NMR (500 MHz, D_2O) δ 4.72 (d, *J* = 8.0 Hz, 1H, H-1I), 4.59 (d, *J* = 7.9 Hz, 1H, H-1II), 4.22-4.18 (m, 3H), 4.04-3.63 (m, 17H), 3.35 (t, *J* = 8.5 Hz, 1H), 2.83 (dd, *J* = 12.7, 4.5 Hz, 1H, H-3eqIII), 1.93 (t, *J* = 12.4 Hz, 1H, H-3axIII); **^{13}C NMR** (125 MHz, D2O) δ 175.8 (CONH), 172.7(COOH), 102.6 (C-1II), 99.2 (C-2III), 95.8 (C-1I), 78.3, 75.5, 75.1, 74.4, 73.8, 72.8, 71.4, 69.4, 68.1, 67.7, 67.6, 62.8, 61.0, 61.0, 60.1, 51.4, 39.3 (H-3III);

α-anomer ^{1}H NMR 500 MHz, D_2O) δ 5.28 (d, *J* = 3.7 Hz, 1H, H-1I), 4.59 (d, *J* = 7.9 Hz, 1H, H-1II), 2.83 (dd, *J* = 12.7, 4.5 Hz, 1H, H-3eqIII), 1.93 (t, *J* = 12.4 Hz, 1H, H-3axIII); **^{13}C NMR** (125 MHz, D_2O) δ 102.6 (C-1II), 99.2 (C-2III), 91.8 (C-1I), 39.3 (C-3III); **HRMS (ESI)**: *m/z* calculated for $C_{23}H_{38}NO_{20}$ [M-H]-, 648.1987; found, 648.2014.
